# Genetic Diversity of Freshwater Leeches in Lake Gusinoe (Eastern Siberia, Russia)

**DOI:** 10.1155/2014/619127

**Published:** 2014-11-27

**Authors:** Irina A. Kaygorodova, Nadezhda Mandzyak, Ekaterina Petryaeva, Nikolay M. Pronin

**Affiliations:** ^1^Limnological Institute, 3 Ulan-Batorskaja Street, Irkutsk 664033, Russia; ^2^Irkutsk State University, 5 Sukhe-Bator Street, Irkutsk 664003, Russia; ^3^Institute of General and Experimental Biology, 6 Sakhyanova Street, Ulan-Ude 670047, Russia

## Abstract

The study of leeches from Lake Gusinoe and its adjacent area offered us the possibility to determine species diversity. As a result, an updated species list of the Gusinoe Hirudinea fauna (Annelida, Clitellata) has been compiled. There are two orders and three families of leeches in the Gusinoe area: order Rhynchobdellida (families Glossiphoniidae and Piscicolidae) and order Arhynchobdellida (family Erpobdellidae). In total, 6 leech species belonging to 6 genera have been identified. Of these, 3 taxa belonging to the family Glossiphoniidae (*Alboglossiphonia heteroclita f. papillosa, Hemiclepsis marginata*, and *Helobdella stagnalis*) and representatives of 3 unidentified species (*Glossiphonia* sp., *Piscicola* sp., and *Erpobdella* sp.) have been recorded. The checklist gives a contemporary overview of the species composition of leeches and information on their hosts or substrates. The validity of morphological identification of each taxon has been verified by phylogenetic approach with a molecular marker adopted for a DNA barcoding of most invertebrates.

## 1. Introduction

**Figure 1:**
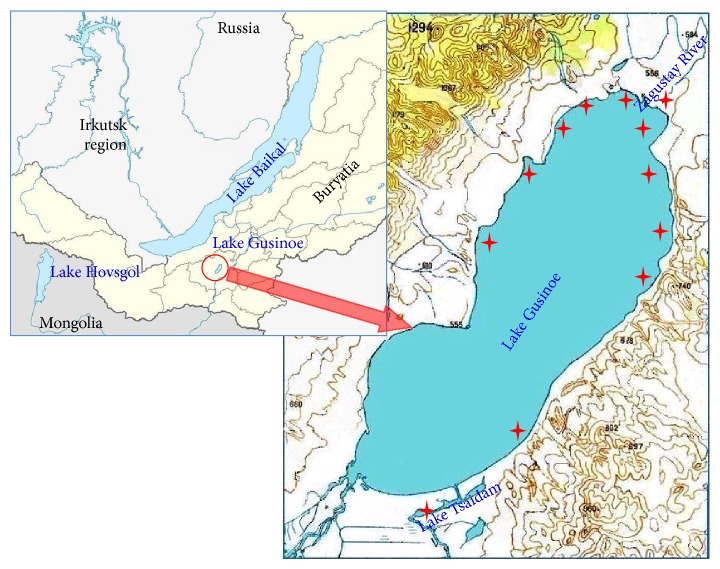
Geographical location of the study region with indication of the main sampling sites in Lake Gusinoe, Lake Tsaidam, and Zagustay River.

Lake Gusinoe located 60 km from the famous Lake Baikal is one of the largest freshwater bodies in the Baikal basin and the largest in the Trans-Baikal region. The origin of this lake goes back to the period of 1740–1749 according to the data collected by the exiled Decembrist Nikolay Bestuzhev. The lake formed as a result of breakthrough of the Temnik River runoff (a tributary of the Selenga) towards the Lake Gusinoe depression and the partial filling of the depression via Lake Tsaidam by Tsagan-Ghol channel [1]. To date, the water area of Lake Gusinoe is 164 km^2^, the volume of water mass is 2.4 km^3^, the length is about 24.8 km, the width is from 5 to 8.5 km, the coastline is 62 km, and maximal depth is up to 28 m averaging 15 m [2]. The catchment area of the Gusinoe basin is 924 km^2^. It has a well-developed fluvial net. In total, there are 72 rivers and creeks with a total length of 312 km [3]. The average density of the river network basin is 0.34 km/km^2^, which is comparable with its great neighbour such as Lake Baikal. The longest tributary of the lake is the Zagustay River. Its length and catchment area are 44 km and 382 km^2^, respectively (Figure 1).

Taxonomic and ecological diversity of the lake biota has been studied insufficiently and unsystematically. Thus, only preliminary information on the existence of 6 Hirudinea species (*Erpobdella octoculata, Glossiphonia complanata, G. heteroclita, Helobdella stagnalis, Hemiclepsis marginata,* and* Piscicola geometra*) was available without the description of their biology and ecology [4]. Furthermore, the species identification was highly doubtful and needed clarification. Meanwhile, leeches are an important part of aquatic biota not only as an element in the trophic level, but also as parasites of other hydrobionts. Interest to Hirudinea sp. has been increased in recent years because of its possible relationship to transmission of bacterial and viral infections [5–9], as well as hematozoa including trematodes, cestodes, and nematodes [10] and parasitic flagellates [11–13], which are considered to be pathogenic organisms for aquatic animals. Moreover, ulceration, hemorrhage, and inflammation associated with leech attachment sites weaken the host undoubtedly and may predispose hosts to bacterial infections.

Sequencing of particular mitochondrial genes in animals, such as Folmer's fragment of cytochrome *c* oxidase subunit I (COI) gene, can yield phylogenetic information and aid in the identification of species. For leeches, DNA barcoding could be particularly useful, as their identification using standard taxonomic techniques can often be ambiguous [14]. In this study, COI sequences of leech species were obtained for specimens collected in different sites of Lake Gusinoe and in eastern Ukraine, resulting in the addition of 14 leech sequences to the GenBank database. Using COI sequences of closely related species that already existed in GenBank, phylogenetic interrelationships were traced out. Genetic diversity was revealed and evaluated. Morphological data were used to corroborate DNA identification.

## 2. Material and Methods

### 2.1. Sample Collection

Biological material was collected during expeditions to Lake Gusinoe in 2012-2013 (Figure 1). Additional samples were collected in the adjacent water bodies, the Zagustay River flowing into the lake in its northern part and Lake Tsaidam adjoining to the southeastern shore of Lake Gusinoe and connecting with the latter by a channel.

To collect leeches, we have inspected various underwater objects (macrophytes, rotten wood, driftwood, snags, stones, etc.) to which hirudinids could be attached. In most cases, piscine leeches were collected directly from the fish caught with different fishing tackles such as fishing net and hydrobiological net or were found on aquatic vegetation (like water milfoil, pondweed, and waterweed).

The leech specimens were directly fixed in 80% ethanol. Alcoholised leech tissues retain flexibility, making investigation of annulation and allocation of genital openings easier. Moreover, such material is suitable for further molecular analysis.

Morphological analysis was performed using a stereomicroscope MSP-2 var. 2 (LOMO). [15–17]. Reference specimens were deposited in the zoological collection of the Laboratory of Molecular Systematics, Limnological Institute.

### 2.2. DNA Sequencing

Sequences of the mitochondrial cytochrome *c* oxidase subunit I (COI) were newly generated from 12 specimens from the Lake Gusinoe area and two individuals of* Glossiphonia concolor* were from Ukraine (Table 1).

Total DNA was extracted from a small portion of the posterior sucker of the leeches according to a slightly modified method using cetavlon [18]. This tissue was selected in order to avoid host blood contamination. COI gene fragments were amplified with oligonucleotide primers universal for most invertebrates [19] using 1.5 *μ*L of primers at 10 *μ*M, 4 *μ*L of dNTP-mix at 10 *μ*M, 0.25 *μ*L of Taq polymerase, 5 *μ*L buffer 10x, 1-2 *μ*L DNA sample, and MilliQ H_2_O for a total volume of 50 *μ*L. Reaction mixtures were preheated to 94°C for 1 min followed by 30 cycles of denaturation at 94°C (30 s), annealing at 47°C (40 s), and extension at 72°C (80 s), and final extension at 72°C (7 min).

PCR fragments were sequenced at the CJSC “Syntol” (Moscow, Russia). The homologous gene sequences of 44 related Hirudinea species available from previous studies were also included in the present analyses for comparative purposes. GenBank accession numbers of all representative species are provided in Table 1.

### 2.3. Bioinformatic Analyses

The sequences were aligned with the ClustalW [20] using default parameters and then were verified by hand. The final dataset matrix included 70 terminals and 709 aligned nucleotide characters. Phylogenetic analyses were carried out using maximum likelihood (ML) method implemented in MEGA V6.06 [21]. All distance values among COI sequences were calculated in MEGA V6.06 using a model of molecular evolution that was considered to describe the substitution pattern the best. Pairwise distance analyses were conducted using the Tajima-Nei model [22]. The differences in the composition bias among sequences were considered in evolutionary comparisons [23]. The percentage of trees in which the associated taxa clustered together was estimated using 500 bootstrap replications. Initial tree for the heuristic search was obtained by applying the Neighbor-Joining method to a matrix of pairwise distances estimated using the maximum composite likelihood (MCL) approach. Comparisons of nucleotide sequences to sequence databases and estimation of the statistical significance of matches, as well as the search for regions of local similarity among the homologous DNA fragments, were performed using the BLAST program [24, 25].

## 3. Results

### 3.1. Taxonomic Review and a Brief Description of Each Taxon

Collection of parasitic and nonparasitic leeches (Hirudinea, Clitellata) from 12 geographical sampling sites (Figure 1) was performed during the two-year seasons in Lake Gusinoe and its catchment area. Morphological analysis revealed six leech species (Table 1: in bold) belonging to two orders (Rhynchobdellida, Arhynchobdellida), three families (Glossiphoniidae, Piscicolidae, and Erpobdellidae) and six genera (*Helobdella*,* Hemiclepsis*,* Glossiphonia*,* Alboglossiphonia*,* Piscicola,* and* Erpobdella*). All six species were found in Lake Gusinoe, and four of them were also detected in Lake Tsaidam, and only two species were reported in the Zagustay River. A poor “catch” in the river was due to the occasional sampling in a limited part of the lower reach of the river.

In the brief commentary, we include a concise description of each leech species with the emphasis on host-parasite relationship, zoogeographical and ecological characteristics, and occurrence of species within the area. Numerical evaluation of biodiversity could be useful for understanding of the importance of species number in terms of actual biodiversity of parasites.

 Phylum ANNELIDA Lamarck, 1809
 Class CLITELLATA Michaelsen, 1919
 Subclass HIRUDINEA Lamarck, 1818 (synonym Hirudinida)
 Order RHYNCHOBDELLEA Blanchard, 1894
 Family GLOSSIPHONIIDAE Vaillant, 1890
 Genus* Hemiclepsis* Vejdowsky, 1884.
(1) *Hemiclepsis marginata (Müller, 1774)* is as follows.
   Local host: Esociformes: Esocidae:* Esox lucius *Linnaeus, 1758. invertebrates.
   Locality: Lake Gusinoe, Lake Tsaidam.

A common Palaearctic species: bloodsucker of fish, tadpoles, and amphipods. Representatives of this species were numerous in the littoral part of these lakes. They were found on stones and in washout from aquatic vegetation. Living leeches are green and brownish-green with a length of 10–12 mm and 2.5–3 mm in width. Alcohol fixed specimens rapidly lose their beautiful intravital colouring.

 Genus* Helobdella* Blanchard, 1876, is as follows.
(2) One considers* Helobdella stagnalis (Linnaeus, 1758).*
   Local host: small invertebrates (oligochaetes, larvae of amphibiotic insects, molluscs, and young amphipods).
   Locality: Lake Gusinoe, Lake Tsaidam.

This species is considered as one of the most common freshwater leeches in the world, a cosmopolite.* H. stagnalis* inhabits shallow coastal parts within Lake Gusinoe and Lake Tsaidam. This is a small but numerous and voracious species. Like all representatives of the family, it shows a touching concern for posterity, bears batch of eggs until their hatching, and then suckles an offspring until its self-dependence.

 Genus* Glossiphonia* Johnson, 1816, is as follows.
(3) One considers* Glossiphonia *sp.
   Local host: Mollusca: Gastropoda.
   Locality: Lake Gusinoe.

For the first time reported in Lake Gusinoe. These leeches were found in a pondweed tangle at a depth of 1.0–2.5 m. Representatives of this group have three pairs of eyes with typical location for the genus, and they are morphologically similar to* G. verrucata*. This leech has a larger body size, reaching 30 mm in length and therefore is the largest representative of the genus in Eurasia.

 Genus* Alboglossiphonia* Lukin, 1976, is as follows.
(4) * Alboglossiphonia heteroclita (Linnaeus, 1761).*

A widespread Holarctic species. This benthic species preys on small invertebrates. There are two forms of this species, which differ in the amount of pigmentation on the dorsal side of the body:* f. papillosa* and* f. striata.* The latter was not found in the study area.

 
*A. * *heteroclita f. papillosa (Braun, 1805).*
   Local host: Mollusca: Gastropoda.
   Locality: Lake Gusinoe, Lake Tsaidam, Zagustay River.

This form is for the first time listed for Lake Gusinoe area. Small-sized glossiphoniids (around 10 mm in length and 3-4 mm in width) have transparent body and are characterized by median row of dark spots. Consider

 family Piscicolidae Johnston, 1865 (=Icthyobdellidae Leuckart, 1863);
 genus* Piscicola* de Blainville, 1818.
(5) * Piscicola *sp. is as follows.
   Local host: Perciformes: Percidae:* Perca fluviatilis* Linnaeus, 1758.
   Locality: Lake Gusinoe.

This leech has been detected for the first time in Lake Gusinoe area. Recently, it has been reported in Lake Baikal [26, 27]. This is a small-sized leech (length up to 8 mm) with a special body coloration differing from a widespread species* Piscicola geometra*. Within Lake Gusinoe, one specimen was found on a perch and another specimen was found in the washout from water milfoil sampled on the north part of the lake. Consider

 Order Arhynchobdellida Blanchard, 1894;
 Suborder Erpobdelliformes Sawyer, 1986;
 Family Erpobdellidae Blanchard, 1894;
 Genus* Erpobdella* de Blainville, 1818.
(6) * Erpobdella *sp. is as follows.
   Locality: Lake Gusinoe, Lake Tsaidam, Zagustay River.

This taxon was recently listed for Lake Baikal [27, 28].* Erpobdella* sp. is widespread within the area. Depending on the environmental conditions, this animal may be a predator of small invertebrates, necrophage, or detritophage. Leeches are of differing sizes. The largest specimen of 56 mm in length and up to 6 mm in width was caught in the mouth of the Zagustay River. Sexually mature individuals with the smallest body size were collected in the north of Lake Gusinoe. Their length and width of the body were 22–28 and 3-4 mm, correspondingly.

### 3.2. COI Phylogeny

**Figure 2:**
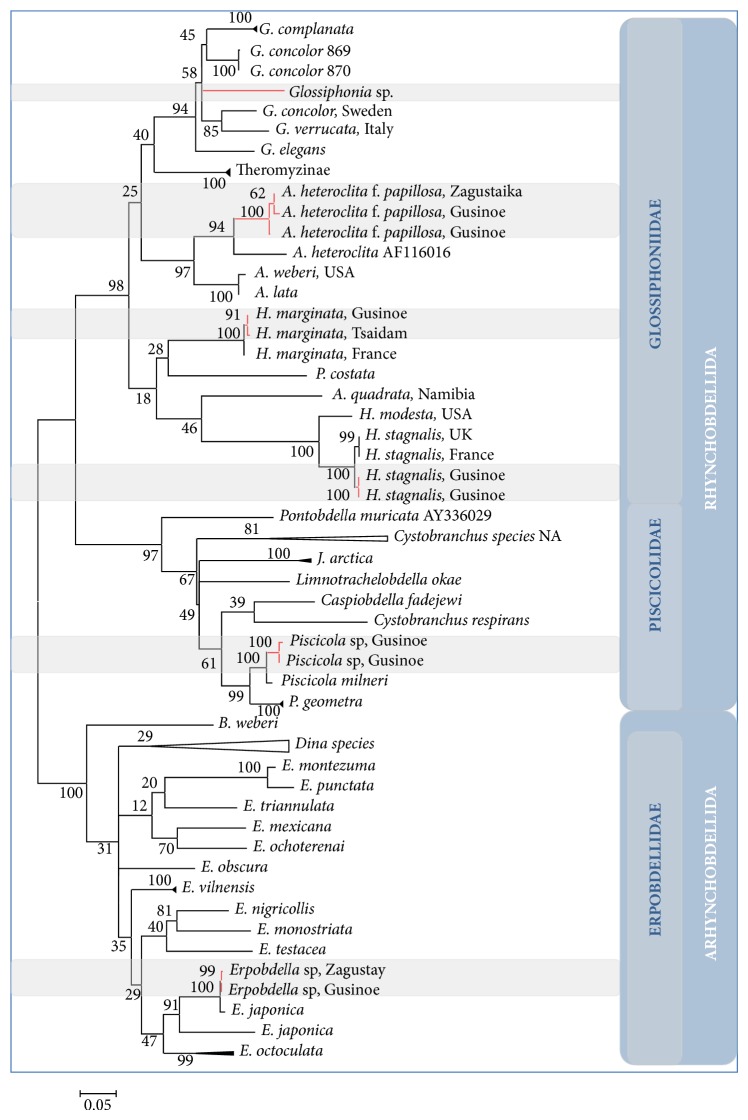
ML-tree based on COI barcoding locus of selected leech species. Lineages of the leeches from the Gusinoe area are highlighted in red. Numbers next to the branches indicate bootstrap values. The tree is drawn to scale, with branch lengths measured in the number of substitutions per site. The analysis involved 70 nucleotide sequences. There were a total of 709 positions in the final dataset.

The final dataset matrix of 70 aligned COI nucleotide sequences in length of 709 base pairs was compiled for phylogenetic analysis. The alignment includes 14 newly generated sequences in this study and 56 other ones closely related to them. All GenBank accession numbers are listed in Table 1. For a reconstruction of phylogenetic interrelationships, one must choose assumptions for modelling of molecular evolution, which are the most suitable for a particular dataset. The evolutionary distance between a pair of sequences usually is measured by the number of nucleotide substitutions occurring between them. The test of the best DNA model for estimating distances was performed in MEGA [21]. As a result, 24 models were suggested. For each model, AICc value (Akaike Information Criterion, corrected), maximum likelihood value (lnL), and the number of parameters (including branch lengths) were also presented. The Tamura-Nei gamma distance with the gamma model taking into account the different rates of substitution between nucleotides and the inequality of nucleotide frequencies (TN93+G+I) was selected for our dataset, since models with the lowest BIC scores (Bayesian Information Criterion) are considered to describe the substitution pattern the best. The Tamura-Nei model [29] corrects for multiple hits, taking into account the differences in substitution rate between nucleotides and the inequality of nucleotide frequencies. It distinguishes between transitional substitution rates between purines and transversional substitution rates between pyrimidines. Nonuniformity of evolutionary rates among sites was additionally modelled by using a discrete gamma distribution (+G, parameter = 0.6761) with 5-rate categories and by assuming that a certain fraction of sites are evolutionarily invariable ([+I], 45.2390% sites). The percentage of trees, in which the associated taxa clustered together, was evaluated using bootstrap analysis. Initial tree for the heuristic search was obtained by applying the neighbor-joining method to a matrix of pairwise distances estimated using the maximum composite likelihood (MCL) approach. The phylogeny was inferred by using the maximum likelihood method based on the chosen model. The tree with the highest log likelihood (−10362.4467) is shown (Figure 2). The tree has a stable topology and a significant statistical reliability of the main branch nodes. The major nodes discussed in the text have the bootstrap value higher than 90%.

The species from the Lake Gusinoe area are clustered in six different lineages according to their generic belonging.

The unclassified jawless leech* Glossiphonia* sp. from Lake Gusinoe forms a separate branch within the species of the genus* Glossiphonia* that corroborates an independent taxonomical status of this species. Genetic distances between* Glossiphonia* sp. and its congeners are above 9.0%, with maximal difference of 12.3% relative to* G. verrucata* (Table 2).

Three Siberian* Alboglossiphonia heteroclita* fall into the common clade with other representatives of the genus. The genetic distances of 13.3% and 13.8% between lineages within the clade distinguish studied samples from* A. lata* and* A. weberi*, correspondingly. There are 11.3% of substitutions accumulated between COI sequences of North Americanand Siberian* A. heteroclita*. The genetic variation within the group of Siberian congeners is about 0.7%.

The representatives of* Hemiclepsis marginata* from France and from the Gusinoe area are grouped together and their COI sequences differ insignificantly (0.6%) that supports the monospecificity of the genus.

The representatives of the Holarctic species* Helobdella stagnalis* form a single lineage with 100% bootstrap support and 0.7% genetic variation within the group regardless of geographical distance of their populations. There is 1.1% genetic distance between European and Siberian specimens. The closest to “stagnalis” clade is* H. modesta* from Ohio, differing from their European and Siberian congeners in 8.6 and 8.4%, correspondingly.

Two piscine leech parasites from Lake Gusinoe are grouped in the same cluster with various forms of the family Piscicolidae (Figure 2). Within the cluster,* Piscicola* sp. appeared more closely related to* P. milneri*, with these two lineages having a genetic distance of 2.3%. Between* Piscicola* sp. and* P. geometra*, there are 7.0% of substitutions in their nucleotide sequences.

Unclassified macrophagous leeches from the Gusinoe area in accordance with their generic belonging clustered within the Erpobdellidae, in close relation to* E. japonica* and* E. octoculata*. The percentage of base substitutions from averaging over all sequence pairs between available* Erpobdella* species groups is shown in Table 3. The resulted genetic distances vary from 4.1% (*punctata*/*montezuma*) to 20.0% (*japonica*/*punctata*). Specimens of* Erpobdella* sp. from Lake Gusinoe and the Zagustay River are the most distant from* E. punctata* (18.1%),* E. triannulata* (16.7%), and* E. mexicana* (16.7%) and, on the contrary, are more closely related to* E. japonica* from Korea (0.7%),* E. japonica* from Japan (10.8%), and* E. vilnensis* (10.7%). All the rest congeners including* E. octoculata* differ genetically from the leeches of Lake Gusinoe by more than 12.1% (Table 3).

## 4. Discussion

### 4.1. Species Diversity

At present, the occurrence of six species in the Lake Gusinoe area has been documented. This species diversity includes both widespread Holarctic and Palaearctic species and also new species from three families and six genera. Three species of the checklist have been reported for the first time in the region, of them* Glossiphonia* sp.,* Piscicola* sp., and* Erpobdella* sp. These leeches were impracticable to determine, since the mosaic set of their morphological characters does not correspond to any known leech species description and currently available systematic keys. Most probably, the leeches with ambiguous species status,* Glossiphonia* sp.,* Piscicola* sp., and* Erpobdella* sp., are potentially new species to science. At the same time,* Piscicola geometra* (Linnaeus, 1761),* Erpobdella octoculata* (Linnaeus, 1758), and* Glossiphonia complanata* (Malm, 1863) were excluded from the species list of Lake Gusinoe. Our precise morphological study of the collected material in the Gusinoe area did not detect these three species.

The first study of Hirudinea was carried out in Lake Gusinoe in the early 1990s [4]. At that time, the species list had included the following species:* Erpobdella octoculata*,* Glossiphonia complanata*,* G. heteroclita*,* Helobdella stagnalis*,* Hemiclepsis marginata*, and* Piscicola geometra*. Until recently, it was assumed that freshwater environments on the vast territory of Siberia had been inhabited by widespread Palaearctic Hirudinea species only. However, the recent targeted studies of lakes and rivers of Eastern Siberia including the unique Lake Baikal [26–28, 30] disprove this hypothesis, since common species* Erpobdella octoculata*,* Glossiphonia complanata*, and* Piscicola geometra* have not been found in the region.

As for the leech* G. heteroclita* mentioned for Lake Gusinoe in the previous study [4], it is necessary to use a scientific binomial name* Alboglossiphonia heteroclita* according to the currently valid systematics of the group. Moreover, the morphological examination allowed us to offer a more detailed determination of the leech taxonomic status, that is,* Alboglossiphonia heteroclita* f.* papillosa*, based on the presence of median row of dark spots on dorsal side, a key characteristic for this form.

Thus, the updated checklist of leech fauna inhabiting Lake Gusinoe consists of* Helobdella stagnalis, Hemiclepsis marginata, Alboglossiphonia heteroclita* f.* striata, Glossiphonia* sp.,* Piscicola* sp., and* Erpobdella* sp.

### 4.2. Genetic Leech Diversity

DNA barcoding is a genomic method used to distinguish between various species of organisms [31]. Since the advent of DNA barcoding, there is widespread speculation that more than 2% of substitutions in marker fragment (COI) seem high enough to suggest the presence of multiple species lineages [31, 32]. Unfortunately, species for which a gene sequence has not been recorded cannot be identified by a database search [33]. The identification of a new species with DNA barcoding and those not included in a genomic repository must be accompanied by a standard taxonomy, geographical information, and other valid species delimitation attributes [34]. Our study has established such DNA barcodes for six species of Hirudinea from the Lake Gusinoe area.

Siberian* Glossiphonia* sp. was found to be related phylogenetically to the relevant species group of the same genus (*G. concolor*,* G. complanata*, and* G. verrucata*, and* G. elegans*). Moreover, nucleotide sequences of Siberian* Glossiphonia* sp. achieved lower similarity values compared to the other* Glossiphonia* species sequences obtained from leeches collected in different European countries (Table 1). Unexpectedly,* Glossiphonia* sp. was genetically far from* G. verrucata* despite their close similarity in external morphology (Table 2). At the same time,* G. concolor* from Sweden was most likely attributed to* G. concolor* erroneously since COI sequences of the reliably identified specimens of* G. concolor* from Ukraine (*G. concolor* 869 and 870, Figure 2) are located on a different branch of the phylogenetic tree. The high level of genetic distinctions, that is, 0.090 of substitutions per site in the barcoding fragment, leads us to consider Swedish and Ukranian “*concolors*” to be in fact two distinct taxa. We have to state that the nucleotide sequence AY962458 does not belong to* G. concolor*.

The molecular data of* Alboglossiphonia heteroclita* obtained from Siberia vary insignificantly within the group (0.7% variable sites). This is a strong support for their species identity. At the same time, 11.3% difference from North American cospecies is too high to be the same species. Consequently, the taxonomic status of leeches with a nucleotide sequence AF116016, which is also used as a DNA barcode, should be verified. Furthermore, there is only 1.0% genetic variation among Asian* A. lata* and American* A. weberi*. This is a convincing evidence of the excessive taxonomic splitting, contrary to the previous conclusion on synonymy of these two species names in favour of* A. weberi* since there are no clear morphological distinctions between* lata* and* weberi* as previously ascertained [15].

Notwithstanding the fact that* Helobdella stagnalis* and* Hemiclepsis marginata* are among the most common leech species, prior to this study, there were only three COI sequences of European representatives available in the GenBank: two* H. stagnalis* and one* H. marginata* (Table 1). The genetic distances between geographically distant representatives of European and Siberian faunas (1.1% and 0.6%, resp.) correspond to a single species in each case.

With regard to piscine leech parasites from Lake Gusinoe, their sequences are more relevant to* P. milneri* and not to* P. geometra*, contrary to the established opinion [4]. However, doubt about species belonging of the Siberian Piscicolidae is still left since the detected genetic distance of 2.3% does not allow them to be confidently attributed to a particular taxon. In this case, an advanced morphological examination of additional material is required.

COI sequences of* Erpobdella* sp. from Lake Gusinoe and the Zagustay River are most genetically close to* E. japonica* from Korea, whereas they vary significantly relatively to* E. japonica* from Japan and* E. vilnensis* (Table 3). From the ML-tree (Figure 2), the* Erpobdella* sp. is found to be a sister taxon of* E. japonica* from Korea with a bootstrap value of 100 for this clade and of* E. japonica* from Japan with a bootstrap support of 92. Genetic difference of 0.7% would allow us with certainty to attribute the unclassified Siberian samples to a certain species, if not a dubious species affiliation of the sample* E*.* japonica* from Korea. The pairwise distance computed between the COI sequence of* E. japonica* from Japan (AB679654) and the sequence of* E. japonica* from Korea (AF116026) was 10.6% of substitutions (Table 3). A high score suggests a significant genetic separateness therefore belonging to distinct species. Since the type specimen for the original description of* E. japonica* was collected in a Japanese lake [35], it is reasonable to recognize the taxonomic designation of the leech specimen (sequence AB679654) as valid, while the Korean sample (sequence AF116026) may be classified in “*japonica*” only* de bene esse*. Unfortunately, the determination of Siberian* Erpobdella* up to species using DNA barcoding is still impossible because it is akin exactly to the incorrectly identified* E*.* japonica*.

In conclusion, at least six leech species occurring in Lake Gusinoe have now acquired DNA barcodes. Our study has shown that a technique of DNA barcoding can be applied successfully to species identification even when morphological taxonomy cannot be employed. High values of genetic distances among species allow considering the fragment of* COI* as a suitable marker for study of inter- and intraspecific relationships in hirudinids. The use of species-specific DNA markers will significantly simplify the identification of Hirudinea species.

## Figures and Tables

**Table 1:** Taxa, localities, information on host species (if known), and GenBank accession numbers for COI sequences of leeches used in phylogenetic analysis.

Species	Collection locality	Host species/substrate	GenBank accession number
Order Arhynchobdellida			
Family Erpobdellidae			
*Dina krilata *	Lake Ohrid, Albania		HM246629
*Dina latestriata *	Lake Prespa, Macedonia		HM246600
*Dina lepinja *	Lake Ohrid, Albania		HM246597
*Dina lineata *	Ligemii Bogovines, Macedonia		HM246611
*Dina lyhnida *	Lake Ohrid, Macedonia		HM246589
*Dina ohridana *	Lake Ohrid, Macedonia		HM24663
*Dina svilesta *	Lake Ohrid, Macedonia		HM246598
*Erpobdella japonica *	Korea		AF116026
*Erpobdella japonica *	Nagano, Japan		AB679654
*Erpobdella mexicana *	Fuentes Brotantes, Mexico		DQ235595
*Erpobdella monostriata *	Mecklenburg-Vorpommern, Germany		HM246601
*Erpobdella montezuma *	Arizona, USA		GQ368760
*Erpobdella nigricollis *	Mecklenburg-Vorpommern, Germany		HM246603
*Erpobdella obscura *	Ontario, Canada		AF003273
*Erpobdella ochoterenai *	Xochimilco, Mexico		DQ235596
*Erpobdella octoculata *	Lake Ohrid, Macedonia		HM246555
*Erpobdella octoculata *	Uzbekistan		HQ336344
*Erpobdella punctata *	Ontario, Canada		AF003275
***Erpobdella* sp.**	**Lake Gusinoe, Russia**	Stones	**KM095091**
***Erpobdella *sp. **	**Zagustay River, Russia**	Stones	**KM095092**
*Erpobdella testacea *	Mecklenburg-Vorpommern, Germany		HM246602
*Erpobdella triannulata *	Catemaco, Mexico		DQ235602
*Erpobdella vilnensis *	Mecklenburg-Vorpommern, Germany		HM246551
*E. vilnensis *	Germany		DQ009663
*E. vilnensis *	Sachsen-Anhalt, Germany		HM246585
Family Salifidae			
*B. weberi *	San Jose, Costa Rica		HQ336339

Order Rhynchobdellida			
Family Glossiphoniidae			
*Placobdella costata *	Italy		AY962461
*Theromyzon bifarium *	North America		AY047330
*Theromyzon tessulatum *	?		AY047318
*Theromyzon pallens *	France		AF003279
*Theromyzon rude *	Ontario, Canada		AF003262
*Hemiclepsis marginata *	France		AF003259
***Hemiclepsis marginata***	**Lake Gusinoe, Russia**	Stones	**KM095093**
***Hemiclepsis marginata***	**Lake Tsaidam, Russia**	*Esox lucius *	**KM095094**
*Helobdella modesta *	Ohio, USA		AF329040
*Helobdella stagnalis *	Cotswolds, UK		AF329041
*Helobdella stagnalis *	France		AF116018
***Helobdella stagnalis***	**Lake Gusinoe, Russia**	Stones	**KM095095**
***Helobdella stagnalis***	**Lake Gusinoe, Russia**	Stones	**KM095096**
***Glossiphonia concolor***	**Ukraine**		**KM095097**
***Glossiphonia concolor***	**Ukraine**		**KM095098**
*Glossiphonia concolor *	Kila Arn, Sweden		AY962458
*Glossiphonia elegans *	North America		AF003258
*Glossiphonia verrucata *	Rio Sadde, Italy		AY962459
***Glossiphonia* sp.**	**Lake Gusinoe, Russia**	Macrophytes	**KM095099**
*Glossiphonia complanata *	United Kingdom		AY047321
*Glossiphonia complanata *	Mecklenburg-Vorpommern, Germany		HM246608
*Glossiphonia complanata *	Aff stream, Paimpont, France		AF003277
*Alboglossiphonia* quadrata	Namibia		AY962455
*Alboglossiphonia lata *	I-Lan County, Taiwan		AY962454
*Alboglossiphonia weberi *	Hawaii, USA		AY962453
*Alboglossiphonia heteroclita *	Michigan, USA		AF116016
***Alboglossiphonia heteroclita***	**Lake Gusinoe, Russia**	Stones	**KM095100**
***Alboglossiphonia heteroclita***	**Lake Gusinoe, Russia**	Stones	**KM095101**
***Alboglossiphonia heteroclita***	**Zagustay River, Russia**	Snag	**KM095102**
family Piscicolidae			
subfamily Piscicolinae			
***Piscicola *sp.**	**Lake Gusinoe, Russia**	*Perca fluviatilis *	**KM095103**
***Piscicola *sp.**	**Lake Gusinoe, Russia**	macrophytes	**KM095104**
*Cystobranchus meyeri *	Tennessee, USA		DQ414315
*Cystobranchus salmositicus *	British Columbia, Canada		DQ414316
*Cystobranchus virginicus *	North Carolina, USA		DQ414317
*Cystobranchus respirans *	Sava River, Slovenia		AY336021
*Johanssonia arctica *	Varangerfjord, Norway	*Paralithodes camtschatica *	AY336012
*Johanssonia arctica *	Newfoundland, Canada		DQ414320
*Limnotrachelobdella okae *	Nevelskoy Strait, Russia	*Huso dauricus *	AY336022
*Caspiobdella fadejewi *	Elz bei Riegel, Germany		AY336020
*Piscicola milneri *	Quebec, Canada		DQ414337
*Piscicola geometra *	Siversky Donets River, Ukraine		AY336015
*Piscicola geometra *	Neckar-Altarm, Germany		AY336014
*Piscicola geometra *	Eyang de la Musse, France		AF003280
Subfamily Platybdellinae			
*Pontobdella muricata *	Gulf of Piran, Slovenia		AY336029

**Table 2:** Estimates of evolutionary divergence (%) over sequence pairs between available *Glossiphonia* species including specimen from Lake Gusinoe.

	*G. complanata *	*G. concolor *	*G. concolor *	*G. verrucata *	*G. elegans *
*G. complanata *					
*G. concolor *	***7.8***				
*G. concolor *	10.1	9.0			
*G. verrucata *	10.9	9.6	8.5		
*G. elegans *	10.7	9.6	***12.3***	***12.3***	
***Glossiphonia *sp.**	**9.0**	**10.1**	**11.8**	***12.3***	**11.6**

**Table 3:** Estimates of evolutionary divergence (%) over sequence pairs between *Erpobdella* species including group of unidentified leeches from the Gusinoe area.

	*E. obscura *	*E. montezuma *	*E. punctata *	*E. triannulata *	*E. mexicana *	*E. ochoterinai *	*E. vilnensis *	*E. nigricollis *	*E. monosriata *	*E. testacea *	*E. octoculata *	*E. japonica *	*E. japonica *
*E. obscura *													
*E. montezuma *	15.8												
*E. punctata *	16.7	***4.1***											
*E. triannulata *	15.2	15.6	16.7										
*E. mexicana *	16.1	17.2	17.7	14.5									
*E. ochoterinai *	13.4	16.1	17.2	14.9	11.6								
*E. vilnensis *	12.1	14.4	15.3	13.5	13.6	12.0							
*E. nigricollis *	14.7	17.4	16.3	14.0	14.7	16.3	11.4						
*E. monosriata *	14.5	16.7	17.0	15.6	16.7	17.9	12.6	10.8					
*E. testacea *	14.7	16.7	18.4	16.1	14.7	16.7	14.0	12.7	14.5				
*E. octoculata *	14.0	16.5	18.2	14.8	16.3	16.4	12.9	12.7	15.4	13.9			
*E. japonica *	14.3	19.8	***20.0***	17.2	17.0	15.8	13.9	14.7	17.2	15.6	14.1		
*E.japonica *	14.9	17.0	18.8	17.0	17.0	16.1	11.4	12.3	14.2	15.4	13.0	10.6	
***Erpobdella *sp.**	**14.5**	**16.3**	**18.1**	**16.7**	**16.7**	**15.4**	**10.7**	**12.1**	**14.3**	**14.7**	**12.8**	**10.8**	**0.7**
